# Locally Advanced Pancreatic Cancer: Percutaneous Management Using Ablation, Brachytherapy, Intra-arterial Chemotherapy, and Intra-tumoral Immunotherapy

**DOI:** 10.1007/s11912-021-01057-3

**Published:** 2021-04-17

**Authors:** Florentine E.F. Timmer, Bart Geboers, Sanne Nieuwenhuizen, Evelien A.C. Schouten, Madelon Dijkstra, Jan J.J. de Vries, M. Petrousjka van den Tol, Tanja D. de Gruijl, Hester J. Scheffer, Martijn R. Meijerink

**Affiliations:** 1 Department of Radiology and Nuclear Medicine, Amsterdam UMC (location VUmc), De Boelelaan 1117, 1081 HV Amsterdam, The Netherlands; 2 Department of Surgical Oncology, Amsterdam UMC (location VUmc), De Boelelaan 1117, 1081 HV Amsterdam, The Netherlands; 3 Department of Medical Oncology, Amsterdam UMC (location VUmc)-Cancer Center Amsterdam, De Boelelaan 1117, 1081 HV Amsterdam, The Netherlands

**Keywords:** Locally advanced pancreatic cancer, Ablation, Radiofrequency ablation, Microwave ablation, Cryoablation, Irreversible electroporation, Brachytherapy, Intra-arterial chemotherapy, Intra-tumoral immunotherapy

## Abstract

**Purpose of Review:**

Pancreatic ductal adenocarcinoma (PDAC) is one of the most aggressive neoplasms, bearing a terrible prognosis. Stage III tumors, also known as locally advanced pancreatic cancer (LAPC), are unresectable, and current palliative chemotherapy regimens have only modestly improved survival in these patients. At this stage of disease, interventional techniques may be of value and further prolong life. The aim of this review was to explore current literature on locoregional percutaneous management for LAPC.

**Recent Findings:**

Locoregional percutaneous interventional techniques such as ablation, brachytherapy, and intra-arterial chemotherapy possess cytoreductive abilities and have the potential to increase survival. In addition, recent research demonstrates the immunomodulatory capacities of these treatments. This immune response may be leveraged by combining the interventional techniques with intra-tumoral immunotherapy, possibly creating a durable anti-tumor effect. This multimodality treatment approach is currently being examined in several ongoing clinical trials.

**Summary:**

The use of certain interventional techniques appears to improve survival in LAPC patients and may work synergistically when combined with immunotherapy. However, definitive conclusions can only be made when large prospective (randomized controlled) trials confirm these results.

## Introduction

Pancreatic ductal adenocarcinoma (PDAC) remains a highly lethal disease, among some of the most challenging neoplasms to treat [1]. Despite advances in systemic regimens (i.e., stronger chemotherapeutics and novel immunotherapies), long-term survival can currently only be achieved through surgical resection of the tumor. However, even then, most patients will develop recurrent disease in the subsequent years, resulting in a 5-year survival of 20% for resected patients [2]. At initial diagnosis, 30–40% of patients present with locally advanced pancreatic cancer (LAPC, stage III) [3]. Herein, LAPC is defined as having > 180° arterial engagement and/or venous involvement, rendering reconstruction unattainable [4]. Although palliative FOLFIRINOX (folinic acid, fluorouracil, irinotecan, and oxaliplatin) is currently the gold standard for stage III tumors, studies have suggested that locoregional treatments may improve overall and disease-free survival [5••, 6, 7•, 8–12]. Percutaneous interventional techniques include radiofrequency ablation (RFA), microwave ablation (MWA), cryoablation, irreversible electroporation (IRE), brachytherapy (iodine-125 (^125^I) seed implantation), intra-arterial infusion of chemotherapy (IAIC), and transarterial chemoembolization (TACE).

**Fig. 1:**
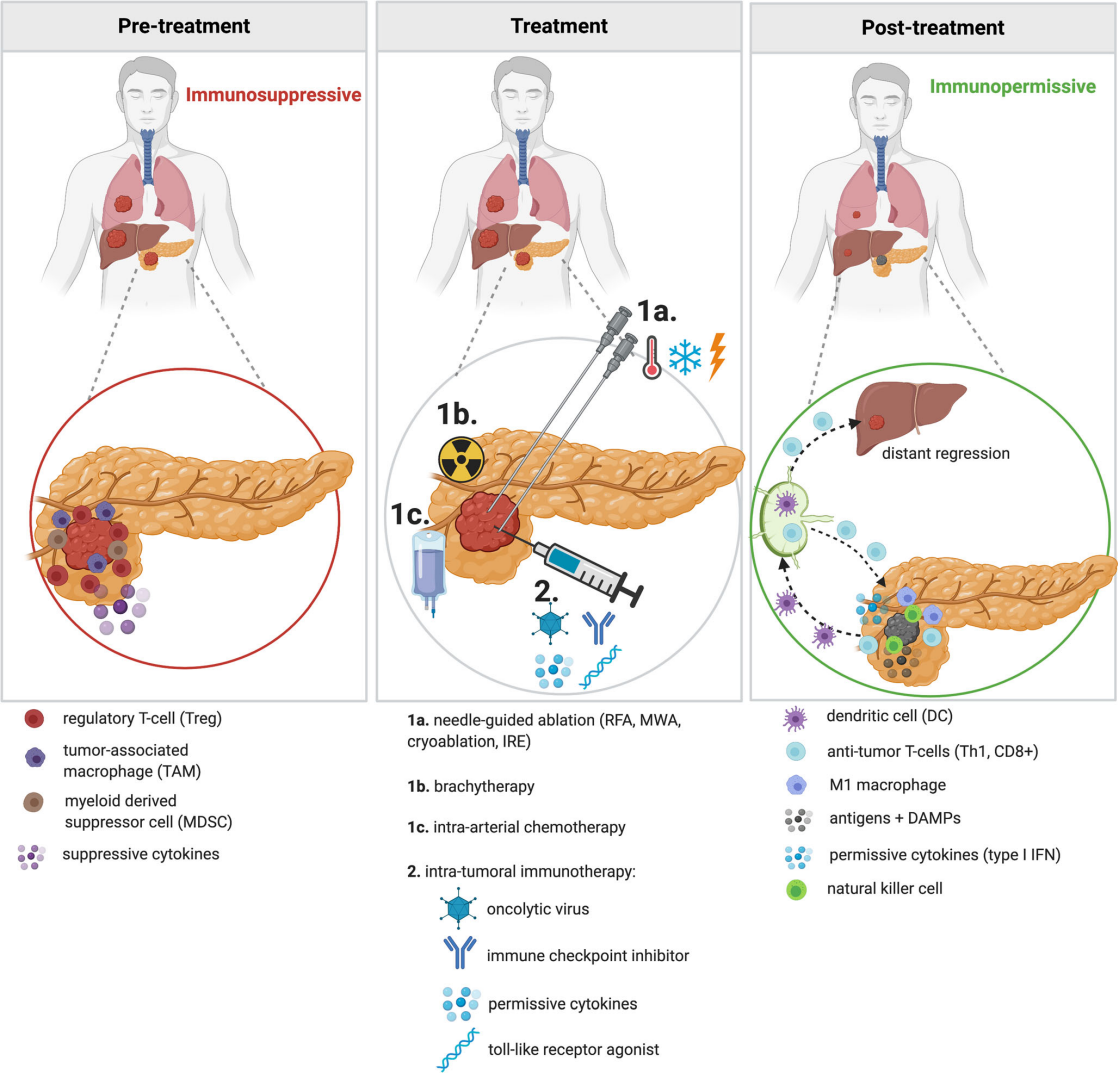
Changing immune status upon treatment with local interventional treatment in combination with local immunotherapy. **Pre-treatment**: pancreatic ductal adenocarcinoma (PDAC) maintains a heavily immunosuppressive environment, established by (among others) regulatory T-cells (Tregs), tumor-associated macrophages (TAMs), myeloid-derived suppressor cells (MDSCs), and suppressive cytokines. **Treatment**: combination treatment with intra-tumoral immune modulation and ablation, brachytherapy, or intra-arterial chemotherapy potentially creates synergism resulting in a durable anti-tumor effect. Immune potentiation combined with local ablation leads to the release of tumor antigens and damage-associated molecular patterns (DAMPs). Subsequently activated dendritic cells (DCs) are now able to capture antigens and migrate towards the draining lymph nodes. Here, antigens are presented to lymphocytes, inducing antigen-specific expansion of effector T-cells, including T-helper-1 cells (Th1) and CD8^+^ (cytotoxic) T-cells, which will provide systemic anti-tumor immunity. Immune activation will lead to reduced TAMs, Tregs and MDSCs. **Post-treatment**: The tumor microenvironment demonstrates a more immunopermissive state, comprising of natural killer cells, M1 macrophages, anti-tumor T-cells (Th1 and CD8^+^), and permissive cytokines such as interferon (IFN). T-cells are also primed to roam the body in search of tumor cells, both at the primary tumor site as well as other locations, possibly resulting in the regression of untreated concomitant (micro)metastases. Figure created with BioRender.com

Another disconcerting feature of PDAC is its highly immunosuppressive tumor microenvironment (TME), established by, among others, the presence of regulatory T-cells (Tregs), myeloid-derived suppressor cells (MDSCs), and tumor-associated macrophages (TAMs) (Fig. 1), limiting the efficacy of chemo- and immunotherapies. In addition to their cytoreductive abilities, ablative, radiotherapeutic, and certain chemotherapeutic strategies have demonstrated immunomodulatory properties by inducing immunogenic cell death (ICD) [13, 14]. The destruction of tumor tissue releases antigens (i.e. proteins of mutated genes) and damage-associated molecular patterns (DAMPs), which are host biomolecules (e.g., DNA, RNA, cytokines) that promote and exacerbate an inflammatory response. These products promote activation of antigen-presenting cells (APCs) such as dendritic cells (DCs), which will subsequently transport antigens to the draining lymph nodes. Here, activation of anti-tumor-specific T-cells is established, theoretically capable of inducing a systemic anti-tumor response in which secondary, non-locally treated tumors display regression [15]. The immune response may be leveraged by combining these tumor-destructive interventional techniques with immunotherapy. This review will focus on locoregional management of LAPC using ablative techniques, internal radiation (brachytherapy), intra-arterial chemotherapy, and intra-tumoral immunotherapy.

## Locoregional Ablation, Brachytherapy, Chemotherapy, and Immunotherapy

Literature from the last decade (2010–2020) describing minimally invasive approaches (i.e., percutaneously or using an endoscope) of ablative techniques (RFA, MWA, cryotherapy, and IRE), brachytherapy (iodine-125 seed implantation), intra-arterial administration of chemotherapeutics (IAIC and TACE), and intra-tumoral immunotherapy is discussed. Specifically, safety and efficacy of these approaches (Tables 1 and 2) are addressed along with their immunomodulatory abilities.

**Table 1:** Studies on ablation in locally advanced pancreatic cancer. Ablation techniques include radiofrequency ablation (RFA), microwave ablation (MWA), cryoablation and irreversible electroporation (IRE). *NS* not specified, *US* ultrasound, *CT* computed tomography, *R* retrospective, *P* prospective, *FFX* FOLFIRINOX, *SAE* serious adverse event, *mOS* median overall survival from diagnosis (mOSd) or treatment (mOSt)

Authors	Year, design	Technique(s)	Approach, image-guidance	Number of LAPC patients	Severe morbidity	Mortality	Survival (months)
Arcidiacono [28]	2012, P	Cryo-RFA	Endoscopic, US	22	0%	0%	mOS 6
D’Onofrio [16]	2017, P	RFA	Percutaneous, US	18	0%	0%	mOS 6
Crino [27]	2018, P	RFA	Endoscopic, US	8	0%	0%	NS
Scopelliti [29]	2018, P	RFA	Endoscopic, US	17	0%	0%	NS
Carrafiello [17]	2013, R	MWA	Percutaneous/open (5/5), US and CT	10	20%	0%	1 yr = 80%
Ierardi [18]	2018, R	MWA	Percutaneous, US and CT	5	0%	0%	NS
Vogl [19]	2018, R	MWA	Percutaneous, CT	22	0%	0%	NS
Niu [50]	2012, R	Cryoablation	Percutaneous, US and CT	11	0%	0%	mOS 12.6 (incl. stages II, III, and IV)
Xu [12]	2013, NS	Cryoablation + brachytherapy (^125^I seeds)	Percutaneous, US and CT	49	6%	0%	mOS 16.2
Dunki-Jacobs [20]	2014, P	IRE	Percutaneous/open (12/53), US	65	NS	NS	NS
Belfiore [21]	2015, R	IRE	Percutaneous, CT	29	0%	NS	MOSt 14
Månsson [22]	2016, P	IRE	Percutaneous, US	24	13%	0%	MOSd 17.9 MOSt 7
Scheffer [6]	2017, P	IRE	Percutaneous, CT	25	40%	0%	MOSd 17 MOSt 11
Narayanan [11]	2017, R	IRE	Percutaneous, CT	50	20%	0%	MOSd 27 MOSt 14.2
Zhang [23]	2017, P	IRE	Percutaneous, US and CT	21	0%	0%	NS
Leen [7•]	2018, R	IRE	Percutaneous, CT	75	7 SAE in 75 pts	0%	MOSt 27
Sugimoto [24]	2018, P	IRE	Percutaneous/open (4/4), US	8	38%	0%	MOSd 24 MOSt 17.5
Ruarus [5••]	2019, P	IRE	Percutaneous, CT	40	21 SAE in 50 pts	4%	MOSd 17 MOSt 9.6
Månsson [25]	2019, P	IRE	Percutaneous, US	24	25%	4%	MOSd 13.3
Flak [26]	2019, P	IRE	Percutaneous, US	33	8 SAE in 33 pts	5%	MOSd 18.5 MOSt 10.7
Van Veldhuisen [10]	2020, R	FFX + IRE vs. FFX	Percutaneous, CT	52	NS AE or SAE	0%	mOSd FFX + IRE 17 FFX 12.4

**Table 2:** Studies on internal radiation (brachytherapy) and intra-arterial chemotherapy in locally advanced pancreatic cancer. Brachytherapy employs iodine-125 seeds. Intra-arterial chemotherapy techniques include IAIC and TACE. *NS* not specified, *R* retrospective, *P* prospective, *CT* computed tomography, *CTA* CT angiography, *IAIC* intra-arterial infusion chemotherapy, *TACE* transarterial chemoembolization, *mOS* median overall survival, *SAE* serious adverse event, *UAP* upper abdominal perfusion

Authors	Year, design	Technique(s)	Approach, image-guidance	Number of LAPC patients	Severe morbidity	Mortality	Survival (months)
Zhongmin [27]	2010, P	Brachytherapy (^125^I seeds)	Percutaneous, CT	10	0%	0%	mOS 7.3 (incl. stages II, III, and IV)
Liu [8]	2015, NS	Brachytherapy (^125^I seeds) ± TACE	Percutaneous, CT	26 (incl. stages III and IV)	NS	NS	mOS 17.6 (stage III)
Yang [28]	2016, P	Brachytherapy (^125^I seeds)	Percutaneous, CT	18	17%	0%	mOS 7.3
Lv [29]	2017, R	Brachytherapy (^125^I seeds)	Percutaneous, CT	32	Grade 2 +: 23%	1.3%	NS
Liu [9]	2012, R (syst rev)	IAIC vs. Syst chemo	Catheter, NS	298 (incl. stages III and IV)	SAE NS	0%	mOS: IAIC 5–21 Syst chemo 2.7–14
Liu [30]	2016, R	IAIC	Catheter, NS	235	NS	NS	mOS 7 (incl. stages III and IV)
Rosemurgy [31]	2017, NS	IAIC	Catheter, CTA	20	10 SAE in 20 pts	0%	1 yr 60% 2 yr 43%
Aigner [32•]	2019, R	IAIC ± embolization vs. UAP	Catheter, NS	174	NS AE or SAE	0%	mOS: IAIC 8 UAP 12
Qiu [33]	2019, R	IAIC	Catheter, NS	31	1%	0%	mOS 5.3

### Radiofrequency Ablation

RFA employs high-frequency alternating currents that create heat, achieving temperatures between 60 and 100 °C, resulting in acute lethal hyperthermia without an excessive increase in impedance [34]. In principle, the extent of cellular damage depends on three factors: amount of energy applied, rate of the energy delivery, and the tissue’s sensitivity to thermal damage. Lethal hyperthermia (> 50–60 °C) induces tissue coagulation and protein denaturation in the central zone, in close proximity to the needle electrode. In the periphery, (sub)lethal temperatures (< 40–50 °C) may result in a combination of necrosis, apoptosis, or recovery, depending on the exposure time [35]. Commonly, one needle electrode is used which is placed into the tumor core, either during laparotomy or laparoscopy, percutaneously, or endoscopically.

#### Efficacy and Safety of RFA

Within the management of pancreatic cancer, endoscopic ultrasound (EUS)–guided RFA has been successfully used for pain palliation by targeting the celiac ganglion [36]. However, the additive value of cytoreductive RFA in the context of LAPC remains controversial, regardless of the utilized method (i.e., open [37–43], percutaneous [16], endoscopic [44–46]). Literature on minimally invasive (percutaneous or endoscopic) RFA is scarce. Three articles employed EUS and one utilized a percutaneous approach. With a median overall survival (mOS) of 6 months from cytoreductive RFA, outcome was poor. Compared to the open approach, these methods demonstrated significantly reduced morbidity (0% vs. 53%) and mortality (0% vs. 6%). D’Onofrio et al. reported on the only percutaneous series of patients with unresectable PDAC (*n* = 18), all of whom were pre-treated with chemotherapy [16]. Technical success was achieved in 16 patients (93%), mOS was 6 months, and none of the patients experienced major complications. Most frequently reported severe complications for pancreatic RFA include pancreatitis, biliary injury, portal vein thromboses, pancreatic fistulas, hemorrhages, duodenal perforations, and gastric ulcers or fistulas [47]. The positioning of the pancreas, entwined with delicate structures, complicates the utilization of thermal ablative methods. For example, the heat-sink effect of adjacent large blood vessels can hinder successful RF ablation. Also, to ensure minimal collateral thermal damage, a safe distance to delicate surrounding parenchyma (i.e., blood vessels and duodenum) must be maintained [48]. This can be minimized by constant intra-procedural cooling of such structures. Ambiguity regarding procedure regimens (temperature, power, duration, minimum distance to vulnerable structures) and timing prior to or after chemo(radio)therapy remains [40, 41]. The currently recruiting randomized controlled PELICAN trial aims to determine the survival benefit of chemotherapy and cytoreductive RFA compared to sole chemotherapy (NCT03690323).

#### Immunomodulation After RFA

The immunomodulation presented after RFA is a result of ICD through the release of antigens and DAMPS such as interleukin (IL)-1, IL-6, IL-8, and tumor necrosis factor (TNF)-α [35]. Specific for thermal tissue damage is the release of a DAMP called heat shock protein (HSP)-70, involved in proper folding and transport of proteins, and believed to play a key role in the immunological response [49]. HSP-70, mainly exposed in the peripheral non-coagulative ablation zone, is elevated in the serum of patients after RFA, which can lead to an immunological anticancer response through activation and maturation of DCs [50, 51]. However, in this peripheral zone, created by diffusion of heat outwards, IL-6, HSP-70, and hypoxia-related pathways have also been implicated to stimulate outgrowth of tumor cells in this area, thus potentially causing (early) recurrences [52–55]. In the coagulative central zone, protein denaturation and destruction of the blood and lymph vessels impedes proper immune infiltration as well as antigen diffusion or transport and subsequent presentation in draining lymph nodes. Clinical evidence of an RFA-induced immune response in PDAC is currently limited to one publication. In this article, Giardino et al. analyzed pre- and post-operative peripheral blood of 10 LAPC patients and found RFA to activate adaptive immune subsets (CD4^+^ and CD8^+^ T-cells) and myeloid DCs, while maintaining stable numbers of immune inhibiting Tregs [56•].

### Microwave Ablation

MWA uses electromagnetic waves to produce tissue-heating. It relies on the oscillation of polar molecules to generate frictional heat, aiming for temperatures between 80 and 150 °C to induce coagulative tissue necrosis [57]. The procedure can be performed using a sole or a cluster of MW antennas which are inserted into the tumor. An advantage of MWA over other thermal ablative techniques includes the faster heating of tissue, making it less susceptible to the heat-sink effect. In addition, MWA appears more suited for larger tumors, is less affected by tissue impedance changes and the microwaves are able to travel more efficiently through fibrous (pancreatic) tissue [58]. However, MWA ablation zones can be harder to predict, possibly leading to overtreatment and, consequently, unintentional thermal damage to adjacent structures. MWA can be performed during laparoscopy, laparotomy, and percutaneously.

#### Efficacy and Safety of MWA

Three groups previously described results following percutaneous MWA in LAPC [17–19]. They included a total of 32 patients treated under US or computed tomography (CT) guidance, mostly focusing on the technique’s feasibility and safety. All reported 100% technical success rates, without any 30-day mortality. Carrafielo et al. included 10 patients (5 percutaneous, 5 laparotomic) with LAPC unresponsive to chemotherapy and were the only group reporting severe morbidity (*n* = 2, 20%) and survival outcomes (1-year OS of 80%) [17]. Major complications included one (early) pancreatitis and one (late) pseudoaneurysm of the gastroduodenal artery. All articles were uniform to conclude the technique’s ability to temporarily improve quality of life, its feasibility, and safety. However, comprehensive and longer term survival results are lacking, thus conclusions on the efficacy of MWA in LAPC are premature. Nonetheless, similar to RFA, a major drawback of this technique remains the use of thermal energy in a highly delicate organ surrounded by vulnerable structures.

#### Immunomodulation After MWA

MWA induces a similar immune response as described for RFA, with upregulation of serum HSP-70, although apparently to a lesser extent [59]. (Pre-)clinical literature on immune modulation of MWA in the context of pancreatic cancer is lacking.

### Cryoablation

With cryoablation, liquid gasses such as argon or nitrogen are delivered through one or multiple cryoprobes and expand into a gaseous state at the tip of the probe through a mechanism known as the Joule-Thomson effect [60]. With temperatures as low as – 190 °C, this process causes local freezing of tissues resulting in a combination of necrotic and delayed apoptotic cell death. Cryoablation depends on four factors: rate of cooling, minimum temperature, and duration at this temperature during the procedure, and the rate of thawing. The extreme cold also induces blood coagulation followed by vascular ischemia [60]. Several freeze-thaw cycles are performed to obtain effective and successful ablation. Cryoablation requires real-time monitoring of the ice ball to ensure complete freezing and minimal injury to adjacent structures.

#### Efficacy and Safety of Cryoablation

To date, four articles have been published on the use of cryoablation in the context of LAPC, two in an open [61, 62] and two in a percutaneous setting [12, 63]. As monotherapy, cryoablation demonstrated a mOS of 12.6 months [63], and in combination with internal radiotherapy a mOS of 16.2 months [12] was noted (neither article specified whether OS from diagnosis or treatment). Niu et al. were the only group reporting on sole percutaneous cryoablation and included patients with stage II (*n* = 3), III (*n* = 11), and IV (*n* = 18) PDAC whose tumor was deemed unresectable [63]. Clinical benefit response (based on pain scores and analgesic consumption) was 84.4%, and the mOS was 12.6 months (incl. all stages). In order to overcome potentially incomplete destruction at the ablation border, Xu et al. described percutaneous cryoablation in combination with brachytherapy in LAPC (*n* = 49), demonstrating a mOS of 16.2 months with a 6% severe complication rate [12]. The freezing process can injure delicate parenchyma including the duodenum, bile ducts, or blood vessels, resulting in inflammation, fistulas, abscesses, or bleeding. Division of surrounding tissue or application of warm pads may reduce the risk of these freezing-related injuries [59].

#### Immunomodulation After Cryoablation

Cryoablation has been described as a potent immunostimulatory inducer among thermal ablative therapies, able to release most non-denatured proteins and induce profound DC antigen loading [64, 65]. It has even resulted in extreme cases of inflammatory responses owing to the induced cytokine storm which increases vascular permeability, resulting in abundant tissue edema and in some cases cryoshock [66]. On the other hand, cryoablation has also been described as an immunosuppressant modality [67]. Increased levels of IL-10, an immunosuppressive cytokine that promotes Treg differentiation, have been proposed as part of the explanation for this [68, 69]. Furthermore, portions of the ablation zone may not exhibit ICD, thus impeding generation of a proper anti-tumor immune response [49]. In this regard, the rate of tissue freezing has been suggested as an important determinant of immunologic response [70]. Literature on the immune response after cryoablation in pancreatic cancer patients is currently limited to one pre-clinical study. White et al. compared early immunological responses after cryoablation and IRE in a rodent model of pancreatic cancer. They noted no significant changes in any immune cell type after cryoablation, compared to a robust intra-tumoral infiltration of macrophages and CD3^+^ T-cells after IRE [71].

### Irreversible Electroporation

**Fig. 2:**
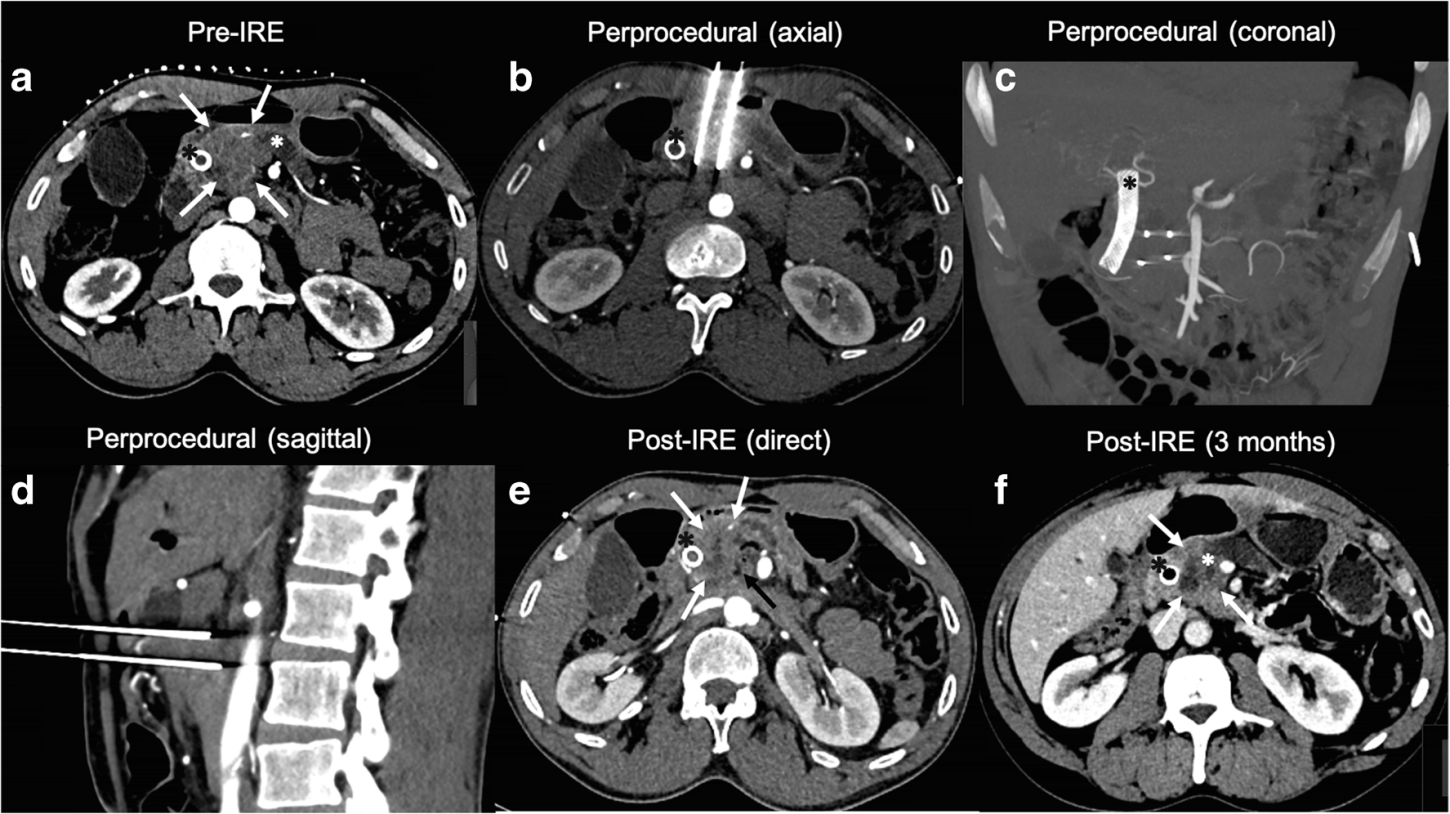
CT-guided percutaneous irreversible electroporation (IRE) for locally advanced pancreatic cancer (LAPC). Sixty-two-year-old male with LAPC on the basis of involvement of the superior mesenteric artery (0–90°, although complete encasement (360°) of the first jejunal branch), involvement of the aorta (0–90°), and involvement of the superior mesenteric vein/portal vein (0–90°). A biliary stent (black asterisks in **a**, **b**, **c**, **e**, **f**) was placed prior to IRE using endoscopic retrograde cholangiopancreatography (ERCP). **a** Perprocedural contrast enhanced (ce)-CT of the LAPC in the head of the pancreas (white arrows) and biliary stent (black asterisk) prior to IRE treatment. The white asterisk shows significant dilation of the pancreatic duct. **b** Perprocedural axial view of 2 of the 4 needle electrodes in situ. **c** Perprocedural coronal view of all 4 needle electrodes in situ. The needles were successfully placed, bypassing all major blood vessels. **d** Sagittal view of 2 of the 4 needle electrodes in situ. **e** ce-CT immediately after IRE. The white arrows delineate the ablation zone, wherein formation of gas pockets is clearly visible (black arrow). The gas pockets maybe the result of water electrolysis and/or vaporization. **f** ce-CT 3 months post-IRE demonstrates a hypointense ablation zone (white arrows). The portal vein is open; dilation of the pancreatic duct (white asterisk) remains unchanged. No evidence of local recurrence or distant metastases

IRE is a predominantly non-thermal ablative technique which utilizes high-voltage electrical pulses (HVEPs) of up to 3000 V to permeabilize and destabilize the cellular membrane, leading to necrotic and (delayed) apoptotic cell death [72]. Multiple needle electrodes are placed in and around the tumor, such that the entire tumor volume including a margin is encompassed. Compared to thermal ablative methods, IRE offers several advantages, especially for pancreatic tumors. The HVEPs preserve the collagen framework of adjacent delicate structures, allowing for cellular regeneration [73]. In addition, the efficacy of the HVEPs is not hindered by the heat-sink effect. For these reasons, US- or CT-guided open IRE, as stand-alone therapy [20, 24, 42, 74–80] or for margin accentuation [76, 81], and percutaneous IRE [5••, 6, 7•, 11, 20–26, 82] have gained interest over the last decade for the treatment of locally advanced or recurrent pancreatic cancer. Figure 2 demonstrates a case of a 62-year-old male with LAPC successfully treated with contrast-enhanced (ce)CT-guided percutaneous IRE.

#### Safety and Efficacy of IRE

Studies on percutaneous IRE demonstrate survival outcomes after diagnosis (mOSd) between 13.3 and 27 months (vs. 15.3–24.9 months in open IRE), and survival outcomes after treatment (mOSt) between 7 and 27 months (vs. 6.4–12 months in open IRE). A large database study has established that IRE in combination with chemotherapy (mOS 16 months) prolongs survival compared to sole chemotherapy (mOS 8 months) after propensity score matching [83]. Leen et al. reported on the largest LAPC cohort (*n* = 75) with the longest survival outcomes (mOSt 27 months). These results may in part be explained by the retrospective nature of the research, inherently leading to selection bias, in combination with favorable patient selection criteria. The largest prospective cohort was reported by Ruarus et al., who treated 40 LAPC and 10 recurrent patients using (ce)CT-guided percutaneous IRE. They reported mOSd and mOSt of 17 months and 9.6 months, respectively. Major complication rates varied substantially among studies on percutaneous IRE (0–40%) but were generally lower compared to open procedures (8–53%). The most frequently encountered major complications during and after IRE procedures include pancreatitis, fistulas, ileus, or delayed gastric emptying, intra-abdominal hemorrhages, ulcers, portal vein thromboses, and biliary-related issues (leakage and/or cholangitis). To reduce the chances of IRE-related infections, prophylactic antibiotics are a must. Reported mortality rates after treatment varied between 0 and 6% (vs. 0–13% in an open setting). Notably, Xu et al. have recently published on the importance of adjuvant chemo- and/or radiotherapy after IRE, concluding that additive adjuvant therapy significantly improves survival [84].

#### Immunomodulation after IRE

Immunomodulation in IRE has been described as the most potent compared to thermal ablative techniques, specifically in terms of protein release and T-cell activation [64]. With IRE specifically, hypoxia is relieved by increasing blood vessel permeability and microvessel density, counteracting hypoxia-induced immunosuppression [85••]. In addition, the preservation of lymph vessels allows DCs to transport released antigens from the ablated area to the draining lymph nodes. Thereafter, the activated effector T-cells are able to infiltrate the residual ablated mass owing to intact blood vessel structures. Scheffer et al. have demonstrated the clinical immune modulatory effects after percutaneous IRE in the peripheral blood of 10 LAPC patients [86•]. Two weeks post-IRE, Tregs were decreased, PD-1^+^ T-cells were elevated, and boosting or de novo induction of a Wilms Tumor-1 (pancreatic TAA)-specific T-cell response was observed. These findings are consistent with a systemic and tumor-specific immune stimulatory effect. Pandit et al. also evaluated the post-IRE immune response in LAPC patients (*n* = 11) [87•], in which three Treg subsets displayed an inverse correlation with time, demonstrating attenuation of immunosuppression.

### Brachytherapy: Iodine-125 Seed Implantation

Iodine-125 (^125^I) seed implantation is a form of brachytherapy, or internal radiation, and has been used in the treatment of PDAC for several decades. The seeds contain the radioactive material and can be placed inside the tumor using an implantation needle and gun [88]. The radioactive material causes direct cell death by damaging its DNA and indirect damage by producing free radicals, resulting in a mixture of lethal and sublethal damage [89]. Since the radioactive particles are embedded inside the tumor and have a small radiation radius, this allows for administration of a high radioactive dose, killing nearby tumor cells without inflicting considerable collateral damage to the surrounding tissue. An important limitation of this treatment is radiation attenuation. With a half-life of 59 days, ^125^I seed implantation will only have a temporary effect. As with the other local therapies, implantation can be accomplished through open [90–94] or percutaneous [8, 12, 27, 28] means under CT or US guidance, with the latter approach having shorter procedural times and quicker recovery [95].

#### Safety and Efficacy of ^125^I Seed Implantation

As stand-alone local therapy, median survival outcomes in LAPC patients percutaneously treated ranges between 7.3 and 11 months. Additional increase in survival can be achieved if ^125^I seed implantation is combined with other interventional modalities such as cryoablation (mOS 16.2 months) [12] or TACE (mOS 17.6 months) [8]. Reported severe morbidity varies between 0 and17% (vs. 0–11% open setting), whereas mortality among studies was 0% (vs. 1.3% open setting). The high rate of severe morbidity reported by Yang et al. (17%) can be explained by patient selection, including only LAPC patients whose disease was complicated by obstructive jaundice [28]. Most common (major) complications for percutaneous ^125^I seed implantation include pancreatitis, fistulas, seed migration, ulcers, infections, leakage, and intestinal perforations [29].

#### Immunomodulation After ^125^I Seed Implantation

Apoptosis is the predominant form of cell death in radiation-based therapies, but high doses of radiation can also lead to necrosis. Similar to other ablative therapies, paradoxical immunomodulatory effects have been reported after radiotherapy treatment [96]. Immunopermissive effects may be initiated by a combinatory release of antigens and DAMPs, followed by activation of the adaptive system. However, radiotherapy has been suggested to promote inactivation of DCs and NK cells, consequently leading to recruitment of immunosuppressive MDSCs and Tregs. There is currently no literature published on the immunomodulatory effects of internal radiation therapy in pancreatic cancer, but external radiation effects were demonstrated by Fujiwara et al. in a subcutaneous PDAC mouse model [97]. Radiotherapy appeared to induce innate immune permissive responses by activating Toll-like receptors (TLRs) and pro-inflammatory chemokines. However, effects on the adaptive system seemed double-edged as immune activating (TNF receptors) and immune suppressive (transforming growth factor (TGF)-β) pathway genes were upregulated. Furthermore, intra-tumoral CD8^+^ T-cells remained scarce.

### Intra-arterial Infusion Chemotherapy/Transarterial Chemoembolization (TACE)

(Regional) intra-arterial infusion of chemotherapy (RAIC or IAIC) and transarterial chemoembolization (TACE) are minimally invasive techniques in which (a combination of) chemotherapeutics are locally supplied through an arterially placed catheter. The latter technique (TACE) includes additional embolization agents to achieve simultaneous blocking of the vessels. IAIC and TACE are often combined with systemic chemotherapy, either in a concurrent or sequential fashion, or used as a subsequent therapy for systemic chemotherapy refractory patients. The chemotherapeutic agents are cytotoxic and non-specifically target either the DNA itself or enzymes required for DNA synthesis and repair, with the rationale that highly proliferative cancer cells are more vulnerable to this damage. IAIC and TACE have been extensively employed for the treatment of hepatic tumors [98], but some literature has also been published on its use in pancreatic cancer [9, 30–33]. The exact tumor location and its supplying arteries determine through which vessels the chemotherapeutic infusion will be delivered. In general, tumors with abundant blood vessels benefit more from regional infusion since this implies a greater localized concentration of the chemotherapeutics [30].

#### Safety and Efficacy of IAIC/TACE

A meta-analysis compared systemic administration of chemotherapy (*n* = 143) with IAIC (*n* = 155) [9]. The analysis comprised 6 randomized controlled trials (RCTs) including 298 patients with advanced pancreatic disease (stages III/IV). They concluded that IAIC resulted in increased median survival (5–21 months vs. 2.7–14 months), superior clinical benefits (78% vs. 29%), and fewer overall complications (49% vs. 71%) and hematological side effects (61% vs. 86%). Although chemotherapeutic toxicity (e.g., neutropenia, thrombocytopenia) rates are lower, they can still arise in IAIC. In addition, specifically with IAIC, (post-procedural) infections or vascular dissections may occur. Although IAIC has shown to be of value, the expansion into common clinical practice is limited by the difficulty of the procedure, with i.v. chemotherapy being more readily available and cheaper. However, especially for patients resistant to standardized systemic chemotherapy, this may be a suitable neo-adjuvant or palliative treatment option.

#### Immunomodulation After (Local) Chemotherapy

(Locoregional) Chemotherapy has been implicated to elicit tumor-specific immune responses [14, 99]. The apoptotic ICD leads to extracellular accumulation of nucleic acids (DAMPs), promoting release of type I interferon (IFN), followed by maturation of DCs and activation of effector T-cells [100]. However, to date no literature has been published specifically describing the immune response after local administration of chemotherapy in pancreatic cancer. Michelakos et al. describe clinical results in PDAC patients (*n* = 248) of whom a portion received systemic FOLFIRINOX (± radiation) and were compared to untreated controls [101]. The treated group exhibited dense CD8^+^ T-cell infiltration, high CD4^+^ T-cell numbers, and low Treg cell density.

### Intra-tumoral Immunotherapy

The aim of immunotherapy is to establish a systemic anti-tumor immune response. Intra-tumoral immunotherapies, in addition to their local priming effects, also enable this systemic effect yet offer one major advantage over systemic administration. Their bioavailability to the tumor and its draining lymph nodes is superior, thus allowing lower doses to suffice and consequently avoiding major systemic toxicities [102–104]. As a result, combination immunotherapies previously deemed unworkable due to severe toxicities are now feasible, with the possibility of repeat treatments [105]. Intra-tumoral infusion can be established under image-guidance, either using an endoscopic or percutaneous (needle-guided) approach. Examples of these intra-tumoral immunotherapies are oncolytic viruses, cytokines, immune checkpoint inhibitors, and Toll-like receptor (TLR) agonists (Fig. 1).

Oncolytic viruses are deployed to infect tumor cells and insert genetic material into their DNA. Oncogenic pathways at play in tumor cells allow for replication of the oncolytic viruses and eventual lysis of the tumor cells, while leaving normal healthy cells unaffected. The lysed tumor cells release tumor antigens, DAMPs, and virus-derived pathogen-associated molecular patterns (PAMPs), leading to immune system activation [106]. Hirooka et al. published results of a phase 1 clinical trial in which LAPC patients (*n* = 10) were treated with EUS-guided intra-tumoral HF-10 oncolytic virus in combination with erlotinib and gemcitabine [107•]. This triple treatment was deemed safe (20% SAEs), with a PFS of 6.3 months and OS of 15.5 months.

IL-12 is a pro-inflammatory cytokine produced by APCs in response to pathogens. It has the ability to induce differentiation of naïve CD4^+^ T-cells into T-helper-1 (Th1) cells, increase cytotoxic activities of T-cells and NK cells, and inhibit or reprogram MDSCs and TAMs [108]. In a PDAC hamster model, an intra-tumoral IL-12 incorporated oncolytic virus achieved a potent anti-tumor effect [109].

Intra-tumoral administration of anti-CTLA4 and/or anti-PD-1 has been shown to ensure optimal access to tumor-draining lymph nodes and in mouse models it has been shown to be equally efficacious as systemic delivery without unwanted immune-related side effects accompanying systemic treatment [102–104]. In a pre-clinical model of PDAC, peri-tumoral anti-CTLA4 has demonstrated effective inhibition of tumor growth, with increased effector T-cell infiltration and reduced Tregs [110].

TLRs are a family of pattern recognition receptors present on the surface of macrophages and DCs, normally involved in the recognition of pathogens. Hereupon, they initiate a cascade of pro-inflammatory effects through the innate and adaptive immune system [111]. Schmidt et al. demonstrated that intra-tumoral administration of a TLR-2/6 agonist in PDAC patients (*n* = 10) with incompletely resected primary tumors resulted in an influx of lymphocytes and monocytes in wound secretion, and reversal of NK inhibition [112].

## Future Directions

The immunomodulatory effects of different treatment strategies are highly variable and have yet to be fully elucidated in the clinical context of PDAC. Nonetheless, a transition from an immune inhibitory to a more permissive state has been presented in several studies. However, one common major limitation remains, i.e., the transient nature of the immune effect. It has been hypothesized that the anti-tumor immune response may become durable when these local techniques are combined with immunotherapy (Fig. 1). In a pre-clinical immunocompetent mouse model with subcutaneous PDAC, stereotactic body radiotherapy (SBRT) in combination with intra-tumoral injections of IL-12 microspheres resulted in increased IFN production and CD8^+^ T-cell activation, followed by significant tumor reduction and even remission in some cases [113]. Zhao et al. utilized a similar PDAC mouse model, demonstrating that combined IRE and systemic anti-PD1 treatment promotes CD8^+^ T-cell infiltration and significantly increased overall survival when compared to the controls and either IRE or anti-PD1 as monotherapy [85••]. Narayanan et al. also presented pre-clinical findings in mice with PDAC, in which IRE was combined with systemic anti-PD1 and an intra-tumoral TLR-7 agonist [114•]. Compared to sole IRE, this triple strategy improved treatment response and resulted in elimination of untreated concomitant metastases. Clinically, IRE combined with NK cells [115, 116•] or allogenic Vγ9Vδ2 T-cell infusion [117••] have both presented as life-prolonging therapies. These encouraging initial results warrant further exploration. The PANFIRE-III trial will combine IRE, systemic anti-PD1, and an intra-tumoral TLR-9 agonist in metastasized PDAC patients (NCT04612530).

## Conclusion

PDAC is an aggressive type of cancer and maintains a highly immunosuppressive environment. The discussed interventional techniques provide cytoreduction of tumor mass in LAPC patients, which may result in prolonged survival. Furthermore, recent literature suggests that certain techniques have immunomodulatory capacities, which may be leveraged when combined with immunotherapy, possibly creating a durable anti-tumor effect. Promising initial data supports this notion of synergism between local interventional and immunotherapeutic strategies. However, definitive conclusions can only be made when large prospective (randomized controlled) trials confirm these results.
